# The Use of γ-Aminobutyric Acid-Producing *Saccharomyces cerevisiae* SC125 for Functional Fermented Beverage Production from Apple Juice

**DOI:** 10.3390/foods11091202

**Published:** 2022-04-21

**Authors:** Xiangyang Sun, Jie Wang, Chanyuan Li, Miaoxin Zheng, Qing Zhang, Wenliang Xiang, Jie Tang

**Affiliations:** College of Food and Bioengineering, Xihua University, Chengdu 610039, China; sunxiangyang95@163.com (X.S.); wangjie196414@163.com (J.W.); lichanyuan795@163.com (C.L.); zhengmmxx9872@163.com (M.Z.); xwllm7687@sina.com (W.X.); wendyjiejie@tom.com (J.T.)

**Keywords:** γ-aminobutyric acid, *Saccharomyces cerevisiae*, fermented beverage, organic acid, volatile compounds

## Abstract

The development of functional fermented beverages enriched with γ-aminobutyric acid (GABA) has been pursued because of the health benefits of GABA; however, few studies have described GABA production by yeast. Therefore, this study aimed to produce fermented apple beverages enriched with GABA produced by *Saccharomyces cerevisiae* SC125. Golden Delicious apples were fermented by *S. cerevisiae* SC125 to produce a novel functional beverage; commercial yeast was used as the control. The GABA, organic acid, and volatile compound content during the fermentation process was investigated by high-performance liquid chromatography and headspace solid-phase microextraction/gas chromatography-mass spectrometry. A yield of 898.35 ± 10.10 mg/L GABA was achieved by the efficient bioconversion of L-monosodium glutamate. Notably, the *S. cerevisiae* SC125-fermented beverage produced several unique volatile compounds, such as esters, alcohols, 6-decenoic acid, and 3-hydroxy−2-butanone, and showed significantly enhanced contents of organic acids, including malic acids, citric acid, and quinic acid. Sensory analysis demonstrated that the *S. cerevisiae* SC125-fermented apple beverage had improved aroma, flavor, and overall acceptability. In conclusion, a fermented functional apple beverage containing GABA was efficiently produced using *S. cerevisiae* SC125.

## 1. Introduction

Fermented beverages are widely consumed worldwide, including fruit wine, yogurt, and vegetable–fruit beverages [1]. Notably, functional fermented beverages have become globally known for their bioactive compounds such as vitamins, probiotics, amino acids, and dietary fiber [2]. Several studies have focused on researching functional fermented beverages, such as selenium-enriched beer [3], probiotic beverages [4], isoflavone enriched soy alcoholic beverages [5], cassava and rice-based beverages with antioxidant activity [6], and bioyogurt from buffalo milk with mannan extract [7].

γ-Aminobutyric acid (GABA) is a non-protein amino acid and is widespread in microbes, animals, and plants [8]. It is the primary inhibitory neurotransmitter in the central nervous system of mammals. In addition, GABA has been shown to lower blood pressure, sedation, and diuresis as well as helping to prevent diabetes [8]. Owing to these health benefits, the development of functional fermented beverages enriched with GABA has been pursued.

It has long been known that lactic acid bacteria (LAB) and yeast are generally recognized as safe (GRAS), and they play important roles in producing fermented beverages. Many articles have reported the use of a single LAB strain to generate GABA-enriched fermented beverages, including black raspberry juice [9], honey syrup [10], and yogurt-like beverages [11]. By contrast, only a few reports have described GABA production by yeasts [8]. *Saccharomyces cerevisiae* is the most common yeast for food fermentation and possesses the qualified presumption of safety status, and many authors have reported its beneficial effects on human health [12]. Thus, utilizing *S. cerevisiae* strains to generate GABA-enriched fermented beverages is of interest. Sichuan paocai, a brine-salted pickle, has a history spanning thousands of years in China, and it is fermented mainly by naturally adherent microorganisms on the vegetable [13]. There are enormously diverse microorganisms that exist during traditional Sichuan paocai natural or spontaneous fermentation. In our previous study, a specific functional yeast strain, named *S. cerevisiae* SC125, was isolated from traditional Sichuan paocai. It has been proven to have a unique capacity for producing GABA, as described by Zhang et al. [14].

Apple is the second most consumed fruit, and it is considered as very rich source of phytochemicals, which may play a role in lowering the risk of chronic diseases [15]. The Golden Delicious apple is one of the most popular apple cultivars globally, especially in the Sichuan-Tibet plateau in China, due to its outstanding quality, high yield, and good flavor [16]. Nevertheless, the species has its disadvantages with storage due to its thin skin and tendency for dehydration compared with other apple cultivars [17]. Accordingly, the production of fermented beverages from Golden Delicious apples can help reduce the level of post-harvest problems. 

Therefore, this study aimed to produce fermented apple beverages enriched with the functional ingredient of GABA. The specific *S. cerevisiae* SC125 was used as a starter culture based on its ability to produce GABA. The pH, ethanol, organic acid, volatile compounds, and sensory properties of the fermented apple beverages were all evaluated. To the best of our knowledge, this is the first attempt to produce a GABA enriched fermented apple beverage using *S. cerevisiae*.

## 2. Materials and Methods

### 2.1. Apple Material

The apple cultivar (Golden Delicious), harvested from a farm in the western Sichuan Plateau of China, was used as a substrate for fermentation. Apple juice was prepared following the methodology of Kaprasob et al. [18]. The apples were cleaned with purified water and cut into small pieces, which were homogenized and blended using a Midea blender (MJ-BL25C4, Guangdong Midea Living Appliances Manufacturing Co., Ltd., Foshan, China) for 5 min. The apple juice was stored at −20 °C until use. After that, the apple juice was pasteurized for 15 min at 70 °C and was used for the fermentation study.

### 2.2. Starter Culture Preparation

*S. cerevisiae* SC125, which was previously isolated from traditional Sichuan paocai, was used as the starter. The yeast was stored at −80 °C at the Xihua University of China in yeast peptone dextrose (YPD) with 5 g/L L-glutamate and 20% (*v*/*v*) sterile glycerol. The yeast was cultured in YPD broth at 30 °C for 24 h, and cells (6 Lg CFU/mL) were used as the starter inoculum. Commercial active dry wine yeast (Hubei Angel Yeast Co., Ltd., Yichang, China) was used as the control.

### 2.3. Fermentation of Apple Juice

The apple juice (250 mL) containing 5 g/L L-monosodium glutamate (L-MSG) was inoculated in duplicate with a final content of 6 Lg CFU/mL starter culture of yeast. The fermentation was performed at 30 °C in 500 mL cotton-plugged and plastic film sealed flasks without shaking for 96 h, and samples were collected every 12 h for analysis.

### 2.4. Analytical Methods

#### 2.4.1. Viable Yeast Cells and pH Analysis

The viable yeast cells were counted using plate counting on a YPD agar at 30 °C for 48 h, and results were presented as Lg-transformed data. The pH of the fermented beverage was determined using a pH meter (pHS−3C, Chengdu Ark Technology Co., Ltd., Chengdu, China).

#### 2.4.2. Analysis of Reducing Sugar, Ethanol, GABA, and Organic Acids

The 3,5-dinitrosalicylic acid (DNS) method was used to determine the reducing sugar content. The ethanol content was assayed using gas chromatography (GC) as described by Liang et al. [19] with slight modifications. The sample was analyzed on a GC system (Agilent Technologies, Inc., Palo Alto, CA, USA), and a DB-Wax column (30 m × 0.25 mm, 0.25 μm film thickness, Agilent Technologies, Inc., Palo Alto, CA, USA) was used, with column and injector temperatures of 60 °C and 200 °C, respectively.

The GABA content was quantified using high-performance liquid chromatography (HPLC) (Waters 2695, Waters corp., Reno, NA, USA) as previously described [14]. The separation column was an Inertsil ODS−3 C18 column (4.6 mm × 150 mm, 5 μm, Shimadzu, Japan). The mobile phases used were A (0.05 M sodium acetate: methanol: tetrahydrofuran, 84: 15: 1, *V*/*V*/*V*) and B (methanol). The flow rate and temperature of the elution phase were maintained at 1.0 mL/min at 30 °C.

The organic acid analyses were also performed using HPLC as previously described [14]. The separation column was an ionic exchange resin Bio-Rad Aminex HPX−87H column (7.8 mm × 300 mm, 9 μm; Bio-Rad Laboratories, Inc., Hercules, CA, USA) with a flow rate of 0.6 mL/min and 5 mmol/L sulfuric acid as the mobile phase. The UV detection wavelength was 210 nm, and the column oven temperature was maintained at 60 °C.

#### 2.4.3. Analysis of Volatile Compounds

The volatile compounds in the fermented beverages were determined using headspace solid-phase microextraction (HS-SPME) and gas chromatography-mass spectrometry (GC-MS), according to the process described by Yang et al. [20] with some modifications (GC temperature program). For GC-MS analysis, each tested sample (4.8 mL) was placed in a 15 mL SPME glass vial with 200 μL internal standard and 2.0 g of sodium chloride. The volatile compounds were semi quantified using 2-octyl alcohol (TCI Chemical Industry Development Co., Ltd., Shanghai, China) as the internal standard.

The vial was tightly sealed and placed in a constant-temperature water bath at 55 °C for 15 min before inserting the aged solid-phase micro extractor into the sample bottle for 30 min. Thereafter, the bottle was pulled out, inserted into the gas chromatograph inlet, and desorbed at 220 °C for 3 min before GC-MS analysis. 

GC-MS analysis was carried out on a Shimadzu GC-MS-QP 2020NX instrument equipped with a DB-Wax column (30 m × 0.25 mm × 0.25 μm film thickness; Agilent Technologies, Santa Clara, CA, USA). GC conditions were as follows: DB-Wax column, injection temperature of 240 °C; temperature program: initial temperature at 50 °C for 2 min, 3 °C/min to 80 °C for 10 min, and 5 °C/min to 230 °C for 6 min; helium as a carrier gas, linear velocity of 1.0 mL/min, and a split ratio of 5:1. Conditions for MS: electron ionization (EI) source, electron energy of 70 eV, filament flow of 0.20 mA, ion source temperature of 200 °C, interface temperature of 250 °C, and scanning range of 30.00–500.00 aum.

### 2.5. Sensory Analysis

Sensory evaluation was carried out in accordance with the method described by Ye et al. [21]. A total of seven sensory indexes, namely aroma, color, flavor, taste, mouthfeel, acidity, and overall acceptability, were chosen to describe the sensory quality and were determined by a hedonic scale of nine categories (1, dislike extremely; 2, dislike very much; 3, dislike moderately; 4, dislike slightly; 5, neither like nor dislike; 6, like slightly; 7, like moderately; 8, like very much; and 9, like extremely). The blind evaluation was carried out in our laboratory by a trained panel of 5 males and 5 females with 20−30 years of extensive experience in cider description. 

### 2.6. Statistical Analysis

All experiments were repeated three times, and the results were expressed as the mean and standard deviation (SD). Origin 8.5 software was used to process the data and plot the figures, SPSS Statistics 22.0 software (IBM) was used for the analysis of variance (One-way ANOVA), and *p* < 0.05 was considered statistically significant.

## 3. Results and Discussion

### 3.1. Viable Cell Growth and pH Profiles during Fermentation

**Figure 1:**
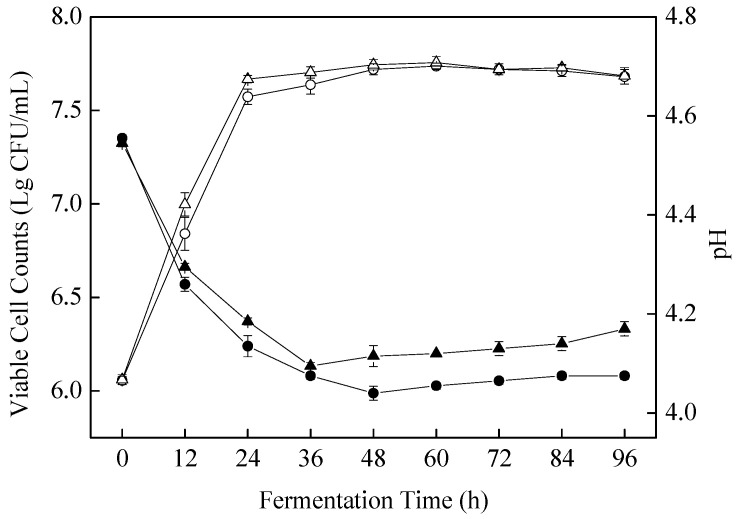
Viable cell counts and pH values were measured during 96 h of apple juice fermentation. The ○ and △ indicate the viable cell counts in *S. cerevisiae* SC125-fermented apple juice and the control. The ● and ▲ indicate pH value of the *S. cerevisiae* SC125-fermented apple juice and the control.

Changes in cell growth and pH value during fermentation by *S. cerevisiae* SC125 in apple juices are presented in Figure 1. There was a significant (*p* < 0.05) increase in the viable cell counts over the apple juices with a fermentation course of 24 h. The cell counts increased slowly and reached the peak value of 7.72 ± 0.03 log CFU/mL at 48 h and remained nearly constant until the end of fermentation. The results suggest that *S. cerevisiae* SC125 growth is well adapted to the apple juice substrate environment.

The change in pH value during fermentation is also depicted in Figure 1. The main role of pH is to confer microbial stability and preserve the sensory properties of beverages [22]. Prior to fermentation, the initial pH value of the apple juice was 4.55; after 48 h of fermentation by *S. cerevisiae* SC125, there was a significant (*p* < 0.05) reduction in the pH value to 4.08 ± 0.01. Compared with the control, the fermentation process using *S. cerevisiae* SC125 resulted in a lower pH value. Notably, previous studies showed that lower pH conditions are beneficial for enhanced GABA synthesis [23].

### 3.2. Changes in Ethanol and Organic Acid Profiles during Fermentation

**Figure 2:**
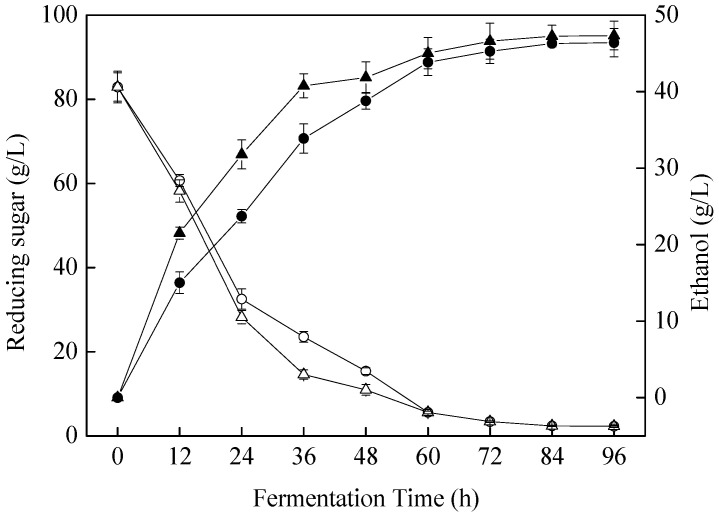
Reducing sugar consumption and ethanol production during 96 h. The ○ and △ indicate reducing sugar consumption in *S. cerevisiae* SC125-fermented apple juice and the control. The ● and ▲ indicate ethanol production in *S. cerevisiae* SC125-fermented apple juice and the control.

Ethanol content is one of the most important indicators for evaluating the *S. cerevisiae* fermentation beverage [24]. As shown in Figure 2, *S. cerevisiae* SC125 showed excellent fermentation performance and produced 46.38 g/L (5.80% *v*/*v*) ethanol from apple juices within 96 h, with a residual reducing sugar content of 2.31 ± 0.17 g/L compared with the control. As the ethanol content was lower than 7% (*v*/*v*), the apple beverage would be classified as a low-alcohol beverage and be in accordance with the healthy drinking trend of today’s society [25].

**Figure 3:**
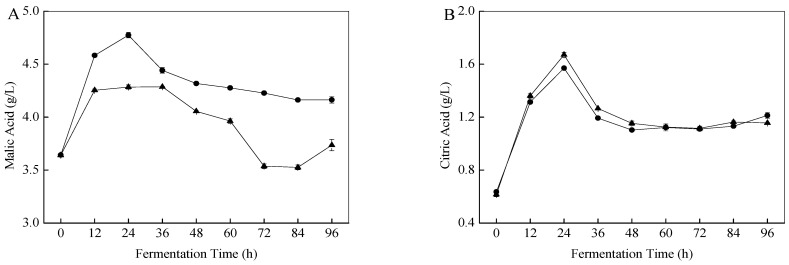

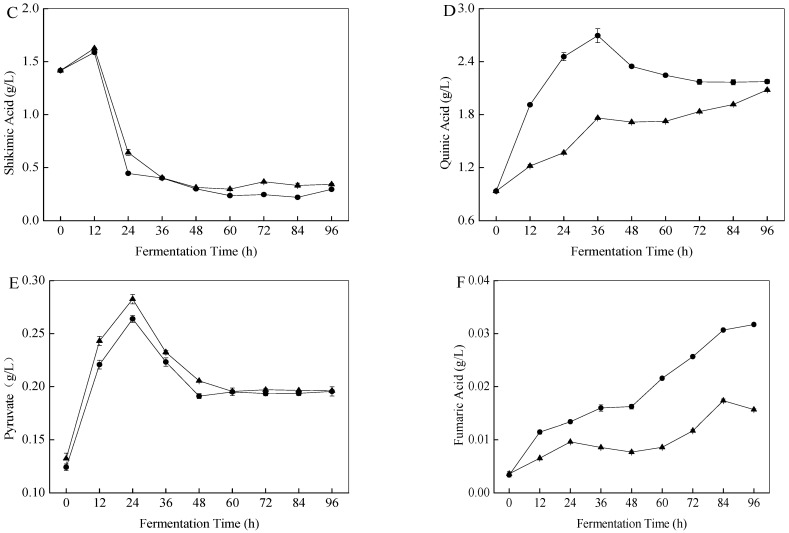
The content of malic acid (**A**), citric acid (**B**), quinic acid (**C**), shikimic acid (**D**), pyruvate (**E**), and fumaric acid (**F**) in *S. cerevisiae* SC125-fermented (●) and control (▲) beverages during the fermentation process.

The organic acids in fermented fruit beverages have a significant impact on the flavor, stability, and quality of the beverages, and they also influence their pH and acceptability [17]. The change profiles of the main organic acids during the apple beverage fermentation process are shown in Figure 3. Malic acid was the most abundant organic acid in apple juice and the fermented beverage (Figure 3A). The initial content of malic acid in apple juice was 3.64 ± 0.01 g/L. Compared with control, the *S. cerevisiae* SC125-inoculated apple juice showed higher malic acid content throughout fermentation, and 4.16 ± 0.03 g/L malic acid was detected after 96 h of fermentation. This could be related to the GABA shunt pathway in *S. cerevisiae* SC125. In yeast, GABA can be catabolized to succinate semialdehyde (SSA) by GABA transaminase, followed by conversion of SSA to succinate, which subsequently feeds into the tricarboxylic acid (TCA) cycle [26]. Succinate then undergoes a series of catalytic reactions to form malic acid. Meanwhile, malic acid can provide a delightful sourness and act as a flavoring agent to create a smoother, more natural taste [27].

Furthermore, citric acid is one of the essential metabolites in the TCA cycle, which provides nutrition and energy sources for microorganisms and promotes cell growth during the fermentation process [28]. Figure 3B shows the curve of citric acid change during fermentation. Citric acid content increased dramatically in both groups during the first 24 h of fermentation and then decreased dramatically until 48 h; 1.21 ± 0.02 g/L citric acid was detected at the end of *S. cerevisiae* SC125 fermentation. Interestingly, the change curves of pyruvate (Figure 3E) and citric acid were similar. Pyruvate is an important metabolite in the Embden-Meyerhof-Parnas (EMP) pathway; it is also a pivotal precursor for many other metabolites, notably citric acid [29]. 

Shikimic acid is one of the major organic acids in apple juice. Upon inoculation with yeast, the shikimic acid content showed a slight upward trend in the first 12 h, followed by a sharp decrease during the next 12 h, and then remained nearly constant until the end of fermentation (Figure 3C). Notably, the decrease in shikimic acid content may be related to the synthesis of quinic acid. It has been reported that the breakdown pathway of shikimic acid can produce 3-dehydroquinic acid, a precursor compound for the synthesis of quinic acid. Thus, this may facilitate an increase in the quinic acid content [30]. In addition, shikimic acid is an essential molecule for the synthesis of aromatic amino acids [30]. The change curve for quinic acid is shown in Figure 3D, with its levels increasing rapidly in the first 36 h after inoculation with yeast. Remarkably, compared with the control, the *S. cerevisiae* SC125-inoculated apple juice showed higher quinic acid content throughout fermentation. Quinic acid has been reported to have anti-vasculitis, anti-neuritis, antioxidant, and radiation protection properties, thus offering many health benefits to consumers [31].

Fumaric acid is the intermediate of the TCA cycle. Figure 3F shows the curve of fumaric acid content change during fermentation. The content of fumaric acid remained extremely low throughout the fermentation period. The results were similar to those of Hranilovic et al. [32], who reported a negative correlation between the content of fumaric acid and ethanol production.

### 3.3. Volatile Compounds Analysis

**Figure 4:**
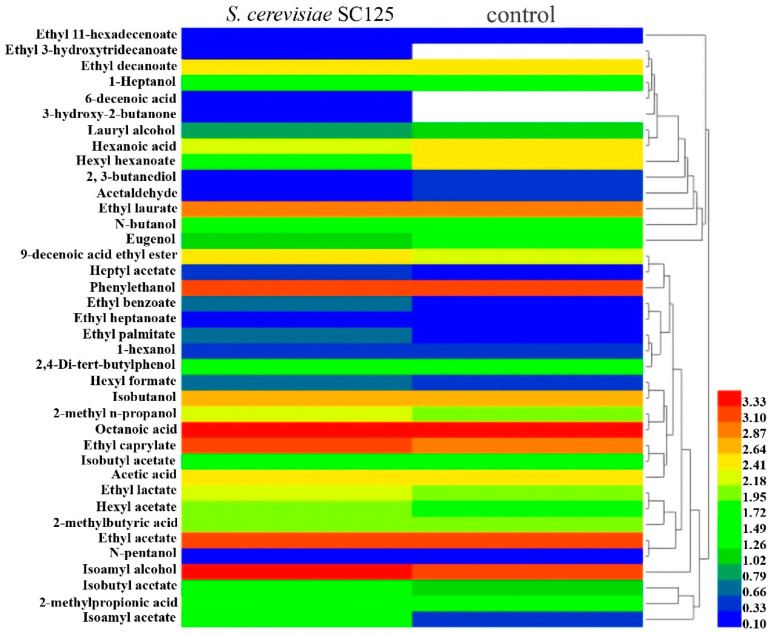
Heatmap of volatile compounds in fermented apple beverages. Different colors indicate different volatile compound content levels. The legend indicates the contents levels from the bottom to the top, where blue colors indicate lower content levels and red colors indicate higher content levels.

Flavor is considered one of the main properties of fermented beverages and an important element to determine the quality of the beverage, which is influenced by the type and content of volatile compounds [33]. Table 1 summarizes the volatile compounds of the two yeasts following the fermentation of apple juice. Overall, the results show significant differences between the different compounds, including esters, alcohols, acids, and other compounds. A heatmap is a very intuitive way to visualize two-dimensional data [34]. To better visualize the flavor differences between the two yeasts fermenting apple juice separately, the heatmap of Figure 4 was utilized to demonstrate the composition and content of volatile compounds, while their contents were transformed by Logarithm. Ethyl caprylate, ethyl acetate, ethyl laurate, isoamyl alcohol, phenylethanol, and octanoic acid were the main compounds shared by the two apple beverages, and ethyl 3-hydroxytridecanoate, 6-decenoic acid, and 3-hydroxy−2-butanone were unique to the *S. cerevisiae* SC125-fermented beverage. The contents of the main compounds, except for ethyl laurate, were higher in the *S. cerevisiae* SC125-fermented beverage than in the non-fermented one. Additionally, the contents of a few other compounds, such as 9-decenoic acid ethyl ester, ethyl lactate, isobutyl acetate, hexyl acetate, 2-methyl n-propanol, and 2-methylbutyric acid, were higher in the *S. cerevisiae* SC125-fermented beverage than in the control. There were similar contents of ethyl decanoate and n-pentanol between the two apple beverages. The results also demonstrated that the *S. cerevisiae* SC125-fermented beverage had lower contents of hexyl hexanoate, lauryl alcohol, 1-heptanol, 2,3-butanediol, hexanoic acid, acetaldehyde, and eugenol than the control. Higher alcohols are considered to contribute positively to the sensory characteristics of the apple cider [35]. The fermentation with *S. cerevisiae* SC125 also produced more alcohols, such as isoamyl alcohol and phenylethanol. 

Importantly, many volatile compounds in fermented beverages have very low sniffing threshold concentrations [36]. Since humans can perceive volatiles with an rOAV > 1, the odor activity values (OAVs; the ratio of the content of the compound to its sniffing threshold) were also calculated for each compound. As shown in Table 1, the volatile compounds with rOAV > 1 in the two apple beverages were ethyl decanoate, ethyl caprylate, and octanoic acid. Additionally, the beverage fermented by *S. cerevisiae* SC125 also contained isoamyl acetate, which is derived from the esterification of acetic acid and isoamyl alcohol, another compound that was present in higher concentrations in beverages fermented by *S. cerevisiae* SC125.

### 3.4. Changes in GABA Production during Fermentation

**Figure 5:**
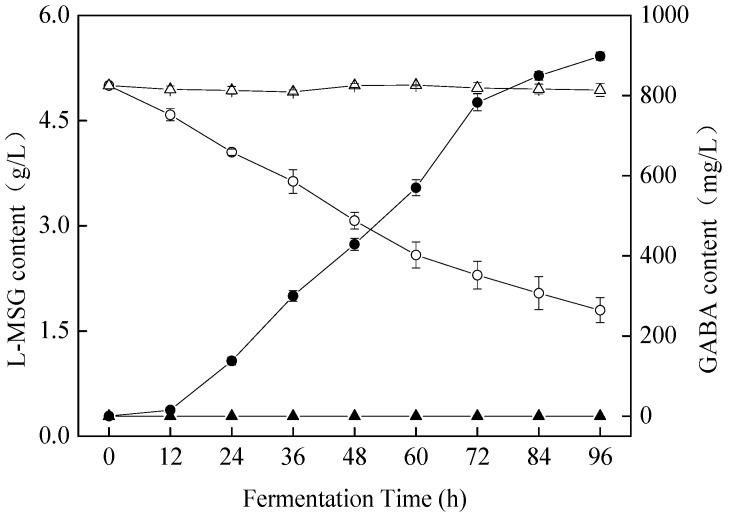
L-MSG and GABA content changes over 96 h. The ○ and △ indicate L-MSG content in the *S. cerevisiae* SC125-fermented and control beverages. The ● and ▲ indicate GABA content in the *S. cerevisiae* SC125-fermented and control beverages.

GABA produced by microbial fermentation has been widely used in the food industry because of its safety and low cost. Consequently, one of the key objectives of this study was to increase the GABA content of apple juice by *S. cerevisiae* SC125 through fermentation. In this study, the GABA content of fermented beverages was analyzed by HPLC, and the quantitative results are shown in Figure 5. The GABA content (898.35 ± 10.10 mg/L) in the fermented beverage described here was greater than other beverages, including grape must beverage (497.97 mg/L) [37], sugar cane juice milk (80.00 mg/L) [38], and mature coconut water (134.00 mg/L) [2]. However, it was lower than black raspberry juice (2.40 g/L) [9]. Previous studies have shown that GABA has stress-relieving effects [8], and 10 mg of GABA in fermented milk administered orally daily was effective for hypertensive patients [14]. Hence, the GABA content in the fermentation beverage described in this report could be sufficient to provide consumers with these health benefits.

### 3.5. Sensory Evaluation

**Figure 6:**
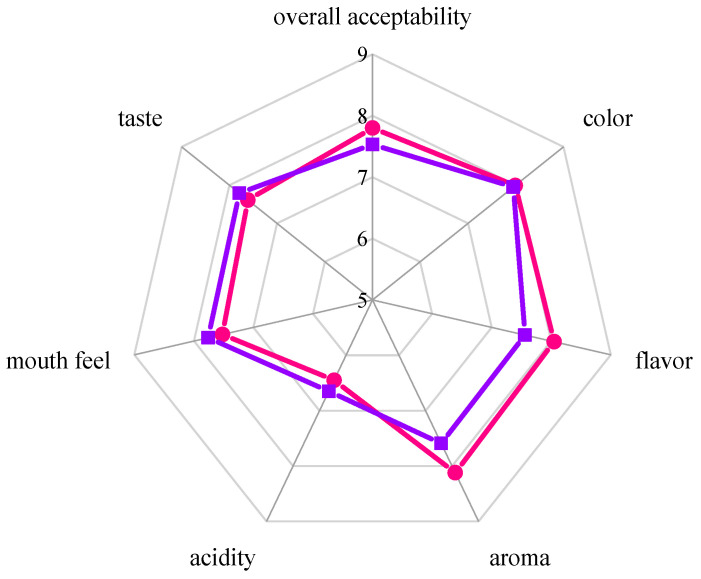
The sensory profile of *S. cerevisiae* SC125-fermented (●) and control (■) beverages.

To evaluate the organoleptic characteristics of fermented beverages, a sensory analysis was performed by a trained panel of 10 judges, using seven evaluation indicators: aroma, color, flavor, taste, mouth feel, acidity, and overall acceptability. Figure 6 depicts the intensity ratings of two fermented beverages after 96 h of fermentation. Both fermented beverages received a high acceptability score. Notably, the beverages fermented with a culture of *S. cerevisiae* SC125 had a higher score for aroma (8.12), flavor (8.05), and overall acceptability (7.80), which may be attributed to the production of more esters and alcohols. 

## 4. Conclusions

The results suggest that Golden Delicious apple juice can be GABA-enriched using *S. cerevisiae* SC125. Furthermore, *S. cerevisiae* SC125 inoculation accelerated the production of organic acids and pH reduction, which is beneficial for enhanced GABA synthesis. Moreover, it massively promoted the formation of volatile compounds—in terms of compound types and content—during fermentation, especially for isoamyl alcohol and isoamyl acetate, thus improving the overall sensory quality of the *S. cerevisiae* SC125-fermented beverage. In conclusion, Golden Delicious apples can be used as a fermentation substrate to create a functional fermented beverage with GABA-producing *S. cerevisiae* SC125. 

## Figures and Tables

**Table 1:** Volatile compound profiles of apple beverages identified by HS-SPME/GC-MS (µg/L).

Compounds	Odor Threshold	Descriptors	Odor Content(μg/L)		OAV	
			SC125	Control	SC125	Control
Esters						
Ethyl 11-hexadecenoate	nf	Buttery	1.49 ± 0.09 ^a^	1.26 ± 0.08 ^b^	-	-
Ethyl 3-hydroxytridecanoate	nf	Nutty	1.49 ± 0.02 ^a^	-	-	-
9-decenoic acid ethyl ester	nf	Fruity	313.47 ± 2.21 ^a^	162.94 ± 3.01 ^b^	-	-
Ethyl benzoate	nf	Fruity	4.48 ± 0.11 ^a^	1.26 ± 0.09 ^b^	-	-
Ethyl heptanoate	nf	Pineapple	1.49 ± 0.21 ^a^	1.26 ± 0.09 ^b^		
Ethyl decanoate	200	Fruity	286.60 ± 2.24 ^a^	286.72 ± 2.29 ^a^	>1	>1
Hexyl hexanoate	nf	Raw fruit	68.67 ± 1.89 ^b^	370.08 ± 3.24 ^a^	-	-
Hexyl formate	nf	Fruity	5.97 ± 0.15 ^a^	2.53 ± 0.01 ^b^	-	-
Ethyl lactate	14,000	Wine	152.26 ± 1.90 ^a^	98.52 ± 0.21 ^b^	<0.1	<0.1
Ethyl caprylate	147	Brandy	1347.93 ± 7.65 ^a^	1139.30 ± 5.11 ^b^	>1	>1
Isobutyl acetate	1600	Pleasant fruity	20.90 ± 0.99 ^a^	11.37 ± 0.23 ^b^	<0.1	<0.1
Heptyl acetate	nf	Rose	2.99 ± 0.08 ^a^	1.26 ± 0.01 ^b^	-	-
Hexyl acetate	1500	Fruity	108.97 ± 2.99 ^a^	73.26 ± 1.31 ^b^	<0.1	<0.1
Ethyl acetate	7500	Slightly fruity	1916.66 ± 5.88 ^a^	1317.39 ± 5.28 ^b^	<0.1	<0.1
Isobutyl acetate	1600	Ripe fruit	29.85 ± 2.29 ^a^	22.74 ± 2.22 ^b^	<0.1	<0.1
Isoamyl acetate	30	Banana, pear	46.27 ± 3.21 ^a^	2.53 ± 0.01 ^b^	>1	<0.1
Ethyl laurate	1500	Flower, fruit	798.61 ± 3.29 ^b^	924.57 ± 4.78 ^a^	>0.1	>0.1
Ethyl palmitate	1000	Weak waxy	4.48 ± 0.11 ^a^	1.26 ± 0.01 ^b^	<0.1	<0.1
Alcohols						
N-pentanol	200	Mellow, astringent	1.49 ± 0.09 ^a^	1.26 ± 0.01 ^a^	<0.1	<0.1
1-hexanol	8000	Grass flavor	2.99 ± 0.11 ^a^	2.53 ± 0.02 ^b^	<0.1	<0.1
N-butanol	5000	Alcoholic	64.19 ± 2.51 ^a^	64.42 ± 1.01 ^a^	<0.1	<0.1
Isoamyl alcohol	30,000	Bitter almond	2268.95 ± 8.90 ^a^	1894.62 ± 9.01 ^b^	<0.1	<0.1
Isobutanol	4000	Fusel alcohol	552.31 ± 2.29 ^a^	492.60 ± 2.11 ^b^	>0.1	>0.1
Lauryl alcohol	1000	Floral fragrance	8.96 ± 0.91 ^b^	11.37 ± 0.09 ^a^	<0.1	<0.1
1-Heptanol	200	Lemon, orange	28.36 ± 1.12 ^b^	37.89 ± 1.01 ^a^	>0.1	>0.1
Phenylethanol	10,000	Rose	1792.77 ± 5.98 ^a^	1447.49 ± 5.21 ^b^	>0.1	>0.1
2-methyl n-propanol	nf	nf	156.74 ± 2.91 ^a^	118.73 ± 0.91 ^b^	<0.1	<0.1
2,3-Butanediol	30,000	Cheese	1.49 ± 0.02 ^b^	2.53 ± 0.01 ^a^	<0.1	<0.1
Acids						
2-methylpropionic acid	nf	nf	52.25 ± 0.99 ^a^	42.94 ± 1.81 ^b^	-	-
2-methylbutyric acid	nf	-	143.30 ± 3.01 ^a^	89.68 ± 1.71 ^b^	-	-
6-decenoic acid	170	Milk	1.49 ± 0.08 ^a^	-	<0.1	-
Hexanoic acid	420	Barbecue flavor	210.47 ± 4.34 ^b^	272.82 ± 2.89 ^a^	>0.1	>0.1
Octanoic acid	500	Fruit flavor	3673.60 ± 9.98 ^a^	2936.65 ± 9.01 ^b^	>1	>1
Acetic acid	4740	Sour	406.02 ± 2.21 ^a^	397.87 ± 1.99 ^b^	<0.1	<0.1
Aldehydes and Ketones						
Acetaldehyde	110	Malt fragrance	1.49 ± 0.01 ^b^	2.53 ± 0.01 ^a^	<0.1	<0.1
3-hydroxy−2-butanone	800	Creamy fragrance	1.49 ± 0.11 ^a^	-	<0.1	-
Phenols						
2,4-Di-tert-butylphenol	nf	Fruity	43.29 ± 1.09 ^a^	20.21 ± 0.90 ^b^	-	-
Eugenol	100	Lilac	16.42 ± 0.31 ^b^	60.63 ± 1.09 ^a^	>0.1	>0.1
TOTAL			14540.66	12315.00		

nf, the threshold of the substance has not been determined. -, not detected. Different superscripts within the same row indicate significant differences at *p* < 0.05.

## Data Availability

No new data were created or analyzed in this study. Data sharing is not applicable to this article.
